# Persuasion effect of corporate social responsibility initiatives in professional sport franchise: Moderating effect analysis

**DOI:** 10.1371/journal.pone.0243579

**Published:** 2020-12-07

**Authors:** Chen-Yueh Chen, Yi-Hsiu Lin

**Affiliations:** 1 Department of Recreation and Leisure Industry Management, National Taiwan Sport University, Guishan, Taoyuan, Taiwan; 2 Master Program of Sport Facility Management and Health Promotion, National Taiwan University, Guishan, Taoyuan, Taiwan; Universitat Jaume I, SPAIN

## Abstract

This study used the elaboration likelihood model as a theoretical basis to explore the effects of various persuasion strategies on consumer perception and attitude regarding the corporate image of sports organizations that engage in corporate social responsibility (CSR) activities. The moderating effects of involvement, sports team identification, and sports fan curiosity were also examined. The multiple-study approach was employed to increase the external validity of the research. Two studies with cross-sectional between-subject pre–post experimental design were conducted with a total of 390 participants. The research setting was the Fubon Guardians baseball team of the Chinese Professional Baseball League in Study I and the Taiwan Beer Basketball Team of the Super Basketball League in Study II. Communication through the central and peripheral routes improved consumers’ CSR perception. Furthermore, under low involvement, weak sports team identification, and low sports fan curiosity conditions, communication through the central route and peripheral route improved the participants’ CSR perception. However, under high involvement, strong sports team identification, and high sports fan curiosity conditions, the different communication methods had nonsignificantly different effects. The findings of this study provide both academic contributions and practical implications.

## Introduction

Corporate social responsibility (CSR) is an issue of perpetual concern for businesses and academicians. The benefits gained by business organizations from CSR input have been verified by numerous empirical studies [1]. CSR activities can satisfy the needs of stakeholders, thereby improving corporate image and business performance [2, 3]. Communication between organization stakeholders plays a vital role in improving corporate image and organizational performance, because consumers’ understanding and perceptions of a company’s social responsibility activities are critical factors [4, 5]. Although companies have boosted their involvement (in terms of focus and resource input) in social responsibility, stakeholders have begun to feel distrustful of the practices or measures adopted by these companies to meet their social responsibility [6]. Therefore, organizations are committed to improving their general corporate image through CSR activities in hopes of meeting the informational need their stakeholders (e.g., consumers) have for knowing that companies are concerned about the interests of the public [7, 8]. Information asymmetry is common and means that consumers are not as well informed as are companies [9]. In two European studies, 75% of German participants believed that they did not have sufficient information on the social or ecological impact of companies’ CSR activity [10], whereas 75% of British participants indicated that consumer buying behavior was affected by greater access to information on organizations’ CSR-related activities [8]. Consequently, communication with stakeholders is crucial for organizations wanting to improve their image and customer loyalty.

Over the past decade, CSR researchers have paid close attention to the communication effects of CSR, and related papers have been published regularly [1, 9, 11–15]. Organizational studies have generated numerous research results relating to the effects of CSR communication; however, research conducted in the sports setting has been scant. Studies relating to social organization issues in sport are mostly focused on (a) the establishment of an integrated framework of CSR in the sports industry [16]; (b) the enhancement of health through sport [17]; and (c) the construction and verification of a scale for measuring consumer attitude toward the CSR of sports organizations [18]. Business in the sports industry are characteristically different to general business organizations, in that consumers of the sports industry identify strongly with and are highly involved in the sporting event or player they support, and their participation in sporting events is highly socially facilitated [19]. Hence, the social responsibility communication of sport-related companies must be extensively explored.

In research investigating the effects of informational communication, the elaboration likelihood model (ELM) has been a key theoretical basis [20]. The ELM proposes two major methods of information persuasion: (a) the central route, in which the message recipient must undergo considerable cognitive information processing; and (b) the peripheral route, in which the message recipient is informed by other fringe factors, such as a spokesperson or the attractiveness of the brand [20–23]. The two routes of information processing in advertising are affected by involvement, ability, and motivation. Despite being crucial components of the information processing mechanism in the ELM, involvement, ability, and motivation are underexplored in CSR communication studies, not to mention sports studies [1]. Numerous scholars investigating the behaviors of consumers in the sports industry have reported that sports team identification consistently influences consumer attitude and behavior [24]. Sports fan curiosity is a major factor promoting sport-related consumption behaviors among sports fans [25]. Hence, this study employed the ELM to examine the moderating effects of involvement, sports team identification, and sports fan curiosity, thereby exploring the mechanism of consumer psychology regarding the effect of CSR communication in sport.

The ELM has long been used in behavioral studies [23, 26]. Several empirical studies have been conducted into CSR communication [1, 11]. However, research has seldom been conducted in the context of sport management’s integration of CSR, which highlights the importance and contribution of the present study. Given the dearth of research in the field of sport management despite the continual development of CSR in both academia and industry, this study employed the ELM as a framework to explore the effects of different persuasion strategies on consumer perception and attitude regarding the CSR image of sports organizations and to examine the moderating influences of involvement, sports team identification, and sports fan curiosity in these effects.

## Literature review

### Conceptualization of CSR

Scholars have varying views on the definition of CSR, with more than 40 definitions being proposed over time [27]. For example, from the social benefit perspective, CSR can be defined as a series of actions taken by companies that are not explicitly required by law and are not based on the interests of the firm but are based on a consideration for the interests of society [28]. In terms of business ethics, CSR can be defined as a type of social obligation, stakeholder obligation, and ethics-driven managerial process [29]. In this perspective, the views of some stakeholders are used as the entry point, but the views of other stakeholders are neglected, potentially creating a theoretical gap [30]. Moreover, from the perspective of stakeholders, CSR refers to a series of social responsibility activities that serve to maintain the relationship between companies and their stakeholders. Although numerous definitions have been proposed, the scope of CSR is none other than an organization’s willingness to improve the well-being of society and stakeholders or avoid damaging society and the environment [31].

### CSR communication

CSR stems from stakeholders having expectations of social responsibility from firms [32]. In recent years, CSR awareness has been widely conceptualized and frequently practiced. However, stakeholder perception of CSR is not entirely positive. For instance, consumers are likely to negatively evaluate the CSR initiatives of sports organizations if they perceive that these organizations engage in CSR activities only for profit rather than in the best interests of society [33]. Furthermore, stakeholder dissatisfaction with a firm’s CSR performance may negatively influence the firm’s corporate image [34]. Therefore, business organizations must effectively communicate with stakeholders to prevent the adverse effects of negative stakeholder perception.

Communicating CSR information to stakeholders is generally difficult for companies [1], because CSR is often communicated through dense printed information [35], and also because CSR messages are extremely complex. For consumers, receiving CSR information involves a mixed-motive information processing task in which the consumer is required to evaluate an organization’s economic and noneconomic performance [9]. In a survey, 70% of consumers found CSR messages difficult to comprehend, leading to questions regarding the effectiveness of CSR communication strategies [1]. Empirical studies have found that the communication of CSR messages has been ineffective, mainly because stakeholders are unaware of companies’ CSR activities [9, 36, 37], believe that companies act in their own interest under the guise of CSR [38], and are skeptical of the earnestness of companies’ CSR activities [39].

Organizations communicate CSR information to stakeholders through social media websites, traditional media, advertisements, direct mail, in-store displays, and product packaging [2, 9, 35]. A study on the CSR of UK supermarkets reported that interactive communication induces a positive perception of supermarket CSR among consumers [14]. Studies exploring perception of CSR indicated that young adults are often targeted because they are regarded as a measure of sociocultural change and are a group of educated and critical consumers, with high levels of social consciousness [14, 40]. Therefore, the present study targeted young adults and selected social media as the medium for information communication, because social media are a form of interactive communication.

Studies examining CSR in sport typically investigate the conceptual framework of CSR and citizenship in sport [16] and provide discourse regarding health promotion by using examples of CSR to promote sport [17]. In sport management, empirical research on CSR mostly revolves around the scope of marketing related to good causes [41–47]. Despite academic efforts made to explore CSR issues in the context of sport, little is known about the effects of CSR communication. In the presented literature analysis, the importance of effective communication between organizations and stakeholders has been emphasized. This is also true for sports organizations. This study therefore explored CSR communication in sport, contributing to the sport management literature on this issue.

### The ELM

This empirical study adopted the ELM as its theoretical foundation. The ELM holds that individuals receiving a message use either the central route or peripheral route to process the message [20]. The central route refers to a high level of relevance of a message to its recipient; when a message has greater personal relevance, the individual processes the message with considerable cognitive effort, meaning that the central route to persuasion mainly involves the processing of attitude information. When individuals process information through the central route, they are likely to diligently consider and evaluate the content of the information. Attitude formation or attitude change induced through the central route is relatively more enduring [48]. By contrast, the peripheral route is characterized by low to nonexistent relevance of the message to its recipient; individuals process the message by relying on heuristic or simple cues such as a spokesperson or expert opinion rather than the main argument of the message. Although attitude formation or attitude change induced through the peripheral route is temporary, unenduring, and not predictive of behavior [20], individuals who process messages through the peripheral route are inclined to activate positive attitudes toward a brand if they feel positive toward a post on social media [49]. Additionally, videos and images can achieve individuals’ engagement on social media [50].

H1: On social media, persuasion through the central route (central-route video and central-route text) and persuasion through the peripheral route (peripheral-route video and peripheral-route image) cause a more positive attitude toward a CSR brand than that of the control group.

### Moderating variables

#### Involvement

Involvement refers to whether an individual perceives an object or decision to be interesting, personally relevant, and important [51, 52]. In the ELM, the effectiveness of different information communication methods is moderated by individual involvement. Numerous empirical studies have indicated that involvement affects the psychological cognition of a person as they process information [48, 53]. Compared with individuals with low involvement, those with high involvement not only exert considerable cognitive effort during information processing [54] but also demonstrate a higher level of cognitive elaboration [11]. Studies have reported that when consumers with higher involvement perceive CSR image, they take more information into consideration, signifying that they elaborate to a greater degree and spend more time processing CSR information [11, 54]. Additionally, empirical studies have indicated that consumers may respond differently to how different firms address their social responsibility; some consumers may identify with a CSR practice and in turn purchase from the firm, whereas other consumers may be indifferent to the firm’s CSR practice. Furthermore, the effects of virtual reality technology on flow experience were greater in sports fans with low involvement than in highly involved sports fans [55]. However, the findings associated with the moderation effect of involvement have been inconsistent; therefore, this study analyzed involvement as a moderator of the persuasion–attitude relationship. Compared with spectators with low involvement, highly involved spectators have a more persistent attitude toward the sports team, which makes them less likely to be affected by persuasion. Therefore, H2 was formed as follows.

H2: On social media, persuasion through the central route (central-route videos and texts) and persuasion through the peripheral route (peripheral-route videos and images) cause a more positive attitude toward a CSR brand than that of the control group among sports spectators with low involvement.

#### Sports team identification

Sports team identification is a concept derived from social identity theory. It refers to an individual’s commitment to a sports team [56]. Social identity theory describes how individuals boost their self-esteem through their connection to a group [57]. Sports team identification extends this concept to an individual’s psychological connection to a sports team formed through his or her identification with the team [58]. Related empirical studies have discovered that a strong attitude results in biased cognitive processing [59, 60]. If individuals are extremely interested in an object (e.g., a sports team), they are inclined to process information according to their initial attitude of support for that object. Additionally, numerous sport management studies have reported that strength of sports team identification influences the cognition, attitude, and consumption behavior of sports consumers [61]. When individuals receive positive information that agrees their initial attitude, they tend to consider this information credible and reliable [62]. Information on a sports organization’s CSR activities is generally related to community development, community involvement, cultural diversity, environmental issues, philanthropy, education, and health [16], which are activities that benefit society, thereby enhancing individuals’ positive attitude toward the sports team. Tormala and Petty also indicated when message recipients are more certain of their attitude toward a target message, biased information processes become evident [62]. In the ELM, the central route is used when a message is highly relevant to its recipient; when a message has greater personal relevance, the individual processes the message with cognitive effort; the central route to persuasion thus mainly involves the processing of attitude information. When individuals process information through the central route, they are likely to diligently consider and evaluate the content of the information. Attitude formation or attitude change induced through the central route is more enduring than that induced through the peripheral route [23].

In addition, individuals who strongly identify with a sports team tend to use media more frequently to follow their sports team [24], are more inclined to purchase team-related merchandise, and spread positive word of mouth for the sports team they support [63, 64]. On the basis of the aforementioned discussion of the literature, this study considered that individuals with strong sports team identification have a more positive attitude toward a brand that communicates CSR information well than those with weak sports team identification. Therefore, H3 was formed as follows.

H3: On social media, persuasion through the central route (central-route videos and texts) and persuasion through the peripheral route (peripheral-route videos and images) cause a more positive attitude toward a CSR brand than that of the control group for sport spectators with low team identification.

#### Sports fan curiosity

Scholars have defined curiosity variously; however, the main implications and spirit of this term are consistent. Litman and Speilberer defined individual curiosity as reaction and desire that induce exploratory behavior [65]. Specifically, curiosity is the desire for exploration to acquire new knowledge or a new sensory experience. Loewenstein defined curiosity as a motivator of individual behavior [66]. In summary, curiosity is a factor driving individuals to acquire new knowledge or experiences.

The role of curiosity in sport can be discussed in terms of the psychological continuum model [19]. This model argues that changing attitude toward a sport, team, or player hinges on several factors, including individual mental state, how knowledgeable the individual is about the sport, socializing agents, and promotional activities held by sports organizations. Funk further elaborated on this model, providing details of four stages: awareness, attraction, attachment, and allegiance [19]. Before individuals become loyal in their sports participation, they first become aware, meaning that they must know about the sport first. After becoming aware of the sport, individuals must begin to like the sport, which is the attraction stage. Between the attraction and allegiance stages is attachment, which is the level of psychological attachment the individuals have to the sport. The four stages of the psychological continuum model are progressive; individuals must first undergo the awareness stage and then continue to engage with or engage in the sport under the influence of media or other medium, after which they develop feelings of likeness for the sport, thus entering the attraction stage. As individuals continue to participate in their favorite sport, they transition to the attachment stage, followed by the allegiance stage. The association between this model and curiosity is that individuals must know about a sport first, and as they enter the attraction stage, their curiosity plays a crucial role [25]. In addition, because curiosity drives individuals to acquire new knowledge and experiences, the stronger an individual’s curiosity, the stronger their need for information. In the ELM, the central route offers a path to gaining relatively abundant information that should meet the informational needs of highly curious individuals. Hence, this study hypothesized that sports fan curiosity moderates the relationship between channel of message persuasion and attitude toward a brand.

H4: Sports fan curiosity moderates the relationship between message persuasion style (central-route video, central-route text, peripheral-route video, or peripheral-route image) and perception of CSR.

## Materials and methods

### Research design and research setting

The multiple-study approach was adopted in this study to increase the research’s external validity. Two cross-sectional studies with between-subject pre–post experimental design were conducted. The research setting was the Fubon Guardians baseball team of the Chinese Professional Baseball League in Study I and the Taiwan Beer Basketball Team of the Super Basketball League in Study II. These teams were selected because they are known for their active participation in CSR activities, which was exactly the requirement of this study. Five experimental treatments were employed, each of which lasted for 1 minute. These five treatments involved central-route video, central-route text, peripheral-route video, peripheral-route image, and a control.

The critical feature that differentiated central- and peripheral-route persuasion in this study was whether participants had diligent consideration in processing the information in the messages. More specifically, central-route persuasion relies on considerable cognitive effort in processing messages, whereas for peripheral-route persuasion, individuals process messages through heuristics or simple cues. Central-route messages included detailed information in 150-word texts and photos pertinent to CSR activities implemented by sports franchises. By contrast, peripheral-route messages used only images as heuristics and simple cues instead of detailed information concerning the CSR activities of sports franchises. Moreover, a 1-min video was created for the central-route scenario in which the 150-word information regarding CSR activities executed by sports teams was verbally described. By contrast, the 1-min peripheral-route video contains information identical to the central-route video but without the 150-word information.

Furthermore, the central-route text had information identical to the central-route video group except that the 150-word information was typed as texts. The peripheral-route image was a 1-min video where all information associated with sports teams’ CSR activities were presented as images without texts and verbal descriptions. Finally, the control group received health care information unrelated to the CSR of a sports team.

Thirty-one college students as participants were recruited to ensure the effectiveness of treatment manipulation for FuBon Baseball Team. They were randomly assigned to one of the four treatments, with 6~9 participants per group (central-route video, central-route text, peripheral-route video, peripheral-route images). All participants were required to rate the statement, “The message requires my diligent consideration in processing its information regarding CSR activities implemented by the sport team,” using a 5-point Likert scale [67] where 1 = disagree strongly and 5 = agree strongly. Independent sample t tests revealed evidence of valid manipulation of the treatment, i.e., central-route text material requires more cognition processing than peripheral-route image one (M_Central Text_= 3.77; SD_Central Text_=1.09; M_Peripheral Image_=1.22; SD_Peripheral Image_=0.66; t=5.98 p<.001). Similarly, central-route video material requires more cognition processing than peripheral-route video one (M_Central Text_= 3.83; SD_Central Text_=0.98; M_Peripheral Image_=2.14; SD_Peripheral Image_=0.89; t=3.23, p=0.008). Moreover, another sample of 28 college students were recruited, and similar results were obtained for Taiwan Beer Basketball Team. Central-route text material requires more cognition processing than peripheral-route Image one (M_Central Text_= 4.14; SD_Central Text_=1.21; M_Peripheral Image_=1.14; SD_Peripheral Image_=0.37; t=6.23 p<.001). Similarly, central-route video material requires more cognition processing than peripheral-route video one (M_Central Text_= 3.14; SD_Central Text_=0.89; M_Peripheral Image_=1.85; SD_Peripheral Image_=0.69; t=3.00, p=0.011).

### Research participants and sampling process

In Study I, the research participants were members of the audience (aged 20 years or older) watching a Fubon Guardians’ game at their stadium. Before the game began, a research assistant asked those entering the stadium whether they were willing to participate in the study. Subsequently, the participants were randomized to one of the experimental treatments. Each experimental group consisted of 40 participants, and 200 participants were recruited in total with 190 valid responses collected. In Study II, the research participants were fans of the Taiwan Beer Basketball Team aged 20 years or older. Because of the coronavirus pandemic, Taiwan Beer Basketball Team played behind closed doors for its 2020 season, which was broadcasted on TV without a live audience. Therefore, fans were recruited online. The principal investigator of this study contacted a professional editor of the Taiwan Beer Basketball Team fan club and asked if any fans were interested in participating in the study. Those who were interested then made contact with the research assistant. Subsequently, the participants were randomized to one of the experimental treatments. Each experimental group consisted of 40 participants, and 200 participants were thus recruited in total (Table 1). After completing the study questionnaire, all participants were gifted a convenience store gift card valued at approximately US$3.30.

**Table 1:** Summary of the participant demographics in this research.

	Study 1 (Baseball, *N*_*1*_ = 190)		Study 2 (Basketball, *N*_*2*_ = 200)	
Variable	*n*	%	*n*	%
**Gender**				
**Male**	105	55.3	115	57.5
**Female**	85	44.7	85	42.5
**Age (years)**				
**21–30**	150	78.9	185	92.5
**31–40**	26	13.7	11	5.5
**41–50**	12	6.3	1	0.5
**51–60**	1	0.5	2	1.0
**>61**	1	0.5	1	0.5
**Education**				
**Junior high school and below**	0	0	1	0.5
**Senior high school**	9	4.7	19	9.5
**Bachelor’s degree**	159	83.7	160	80.0
**Master’s degree and above**	22	11.6	20	10.0

### Research procedures

This study was reviewed and approved by the Institutional Review Board of National Taiwan University. Tests for Study I participants were administered at the stadium. The research assistant first explained to the participants the study objectives before obtaining their consent to participate. After consent was obtained, the participants were added to a LINE app group and then randomized to one of the five experimental treatments. Once the five LINE groups had been established, the participants began completing the questionnaire; their participation was concluded once they had completed the questionnaire. The content and sequential order of the questions were as follows. The participants first detailed their perceptions of the Fubon Guardians’ CSR activities (pretest score) and then their involvement in the baseball team, sports fan curiosity, and team identification. After completing these scales, the participants were asked to read the information provided (which varied according to their experimental group) and tick the box stating “I have finished reading this information” to confirm that they had read it. Next, the participants were asked to detail their perceptions of Fubon Guardians’ CSR activities (posttest score) again. Finally, the participants’ demographic information was collected. The participants were allowed to withdraw from this study unconditionally at any time during the course of the questionnaire. The same testing procedure was administered in Study II, the only difference being the research setting (Taiwan Beer Basketball Team).

### Research instruments

Involvement was measured using the instrument employed by Beatona, Funk, Ridinger, and Jordan [68]. The sports fan curiosity scale was adapted from that of Park, Mahony, and Greenwell [69]. Sports team identification was measured using the scale of Robinson and Trail (2005). Attitude toward CSR brand was measured using the scale of Walker and Heere [18]. The scales were scored using a 7-point Likert scale ranging from 1 (*strongly disagree*) to 7 (*strongly agree*). The demographic data collected were those on sex, age, education level, and occupation.

To ensure the quality of the research instruments in this study, confirmatory factor analysis was used to examine the goodness of fit of each scale. As recommended by Hair et al. [70], the construct validity of the research instruments was verified; standardized factor loadings were greater than .5, which indicates that the model exhibited convergent validity. The reliability of scale dimensions was examined using internal consistency reliability, with the acceptable range generally being .7 or larger (Table 2). The average variance extracted of two factors had to be greater than the square of their correlation coefficient to indicate that the model exhibited discriminant validity. While sub-dimensions within a construct did not meet the criterion, the discrimination across constructs was sufficed (Table 3).

**Table 2:** CFA summary for Study 1 (*N*_*1*_ = 190) and Study 2 (*N*_*2*_ = 200).

Factor/item	*M*	SD	*λ*	*t*
**Attitude toward CSR—Pre-score**				
**Cognitive Awareness [*α* = 0.89 (0.92); AVE = 0.74 (0.82)]**				
**1. I am aware of the social programs of FuBon Baseball Team (Taiwan Beer Basketball Team).**	4.41 (5.05)	1.80 (1.72)	.87* (.89*)	-- (--)
**2. I know of the good things FuBon Baseball Team (Taiwan Beer Basketball Team) does for the community.**	4.30 (4.94)	1.73 (1.77)	.91* (.95*)	17.54 (22.28)
**3. I believe FuBon Baseball Team (Taiwan Beer Basketball Team) to be a socially responsible organization.**	4.76 (5.38)	1.57 (1.51)	.81* (.88*)	14.24 (18.21)
**Affective Evaluation [*α* = 0.93(0.95); AVE = 0.79 (0.86)]**				
**1. I feel good about FuBon Baseball Team (Taiwan Beer Basketball Team) partly because of the things they do to benefit the community.**	4.40 (5.20)	1.58 (1.65)	.86* (.93*)	-- (--)
**2. Part of the reason I like FuBon Baseball Team (Taiwan Beer Basketball Team) is because what they do for the community.**	4.32 (5.04)	1.60 (1.66)	.97* (.95*)	20.73 (26.03)
**3. One of the reasons I speak positively about FuBon Baseball Team (Taiwan Beer Basketball Team) is because of what they do for the community.**	4.35 (5.10)	1.64 (1.61)	.92* (.93*)	18.51 (24.86)
**4. I buy merchandise from FuBon Baseball Team (Taiwan Beer Basketball Team) partly because I believe they are a socially responsible organization.**	4.02 (4.92)	1.70 (1.78)	.80* (.90*)	14.25 (21.61)
**Involvement**				
**Hedonic value [*α* = 0.94 (0.91); AVE =0.83 (0.79)]**				
**1. Baseball (Basketball) is fun.**	5.81 (6.33)	1.40 (1.11)	.85* (.84*)	-- (--)
**2. Baseball (Basketball) is one of the most satisfying things that I do.**	5.44 (6.10)	1.55 (1.26)	.93* (.93*)	18.14 (17.34)
**3. I really enjoy Baseball (Basketball).**	5.26 (5.94)	1.70 (1.48)	.96* (.90*)	19.47 (16.56)
**Centrality [*α* = 0.94 (0.93); AVE =0.85 (0.82)]**				
**1. I find a lot of my life organized around Baseball (Basketball).**	4.46 (5.44)	1.98 (1.69)	.91* (.85*)	-- (--)
**2. Baseball (Basketball) plays a central role in my life.**	4.29 (5.36)	2.09 (1.69)	.95* (.95*)	23.92 (19.53)
**3. I enjoy discussing Baseball (Basketball) with others (e.g., friends, family, co-workers).**	4.57 (5.55)	2.08 (1.66)	.91* (.92*)	21.51 (18.33)
**Symbolic value [*α* = 0.96 (0.95); AVE = 0.90 (0.88)]**				
**1. Baseball (Basketball) says a lot about who I am.**	3.76 (4.62)	2.03 (2.01)	.95* (.94*)	-- (--)
**2. Baseball (Basketball) tells something about me.**	3.83 (4.76)	2.06 (2.04)	.96* (.96*)	30.19 (28.31)
**3. Baseball (Basketball) gives others a glimpse of the type of person I am.**	3.78 (4.87)	2.03 (1.98)	.95* (.92*)	28.78 (24.49)
**Sport Fan Curiosity**				
**Excitement [*α* = 0.84 (0.82); AVE = 0.57 (0.53)]**				
**1. I enjoy fipping through sport network channels when I feel bored.**	4.92 (5.39)	1.58 (1.45)	.77* (.68*)	-- (--)
**2. I enjoy being around “die-hard” sport fans to have a new experience.**	5.36 (5.95)	1.44 (1.19)	.73* (.71*)	10.05 (8.91)
**3. I often look for new opportunities to watch sports.**	4.85 (5.21)	1.52 (1.61)	.77* (.71*)	10.66 (8.93)
**4. I enjoy probing deeply into new sports or leagues.**	5.03 (5.35)	1.50 (1.48)	.76* (.82*)	10.52 (10.05)
**New Sport Events [*α* =0.84 (0.89); AVE = 0.65 (0.74)]**				
**1. I enjoy watching a major sport event for the first time.**	5.61 (6.00)	1.34 (1.19)	.80* (.82*)	-- (--)
**2. Watching new sport events with my friends is exciting.**	5.32 (5.77)	1.47 (1.36)	.80* (.88*)	11.94 (15.24)
**3. My curiosity is aroused when watching exciting new sport events.**	5.85 (6.11)	1.27 (1.19)	.82* (.88*)	12.40 (15.30)
**Sport Facility [*α* = 0.72 (0.74); AVE = 0.46 (0.51)]**				
**1. I enjoy exploring brand new sport stadiums or facilities.**	5.09 (5.68)	1.40 (1.41)	.72* (.84*)	-- (--)
**2. When I see a new sport facility on television, I want to go to it and explore it.**	5.83 (6.06)	1.23 (1.17)	.69* (.77*)	9.70 (13.19)
**3. When visiting a brand new sport facility, I want to explore it.**	4.71 (5.18)	1.50 (1.59)	.63* (.51*)	8.88 (7.82)
**Team Identification [*α* = 0.96 (0.96); AVE =0.91 (0.91)]**				
**1. I consider myself to be a “real” fan of FuBon Baseball Team (Taiwan Beer Basketball Team).**	4.13 (5.04)	1.91 (1.88)	.93* (.91*)	-- (--)
**2. I would experience a loss if I had to stop being a fan of FuBon Baseball Team (Taiwan Beer Basketball Team).**	3.92 (4.65)	2.19 (2.12)	.98* (.99*)	28.95 (27.95)
**3. Being a fan of FuBon Baseball Team (Taiwan Beer Basketball Team) is very important to me.**	3.84 (4.66)	2.23 (2.16)	.96* (.97*)	26.77 (25.91)
**Attitude toward CSR—Post-score**				
**Cognitive Awareness [*α* =0.91 (0.95); AVE = 0.80 (0.87)]**				
**1. I am aware of the social programs of FuBon Baseball Team (Taiwan Beer Basketball Team).**	5.01 (5.69)	1.61 (1.48)	.92* (.95*)	-- (--)
**2. I know of the good things FuBon Baseball Team (Taiwan Beer Basketball Team) does for the community.**	4.91 (5.67)	1.52 (1.49)	.94* (.95*)	22.51 (29.13)
**3. I believe FuBon Baseball Team (Taiwan Beer Basketball Team) to be a socially responsible organization.**	5.12 (5.76)	1.32 (1.36)	.83* (.91*)	16.29 (24.50)
**Affective Evaluation [*α* =0.95 (0.96); AVE =0.84 (0.88)]**				
**1. I feel good about FuBon Baseball Team (Taiwan Beer Basketball Team) partly because of the things they do to benefit the community.**	5.02 (5.70)	1.39 (1.41)	.92* (.95*)	-- (--)
**2. Part of the reason I like FuBon Baseball Team (Taiwan Beer Basketball Team) is because what they do for the community.**	4.84 (5.59)	1.46 (1.45)	.94* (.96*)	23.35 (33.07)
**3. One of the reasons I speak positively about FuBon Baseball Team (Taiwan Beer Basketball Team) is because of what they do for the community.**	4.86 (5.65)	1.39 (1.46)	.94* (.95*)	23.17 (30.06)
**4. I buy merchandise from FuBon Baseball Team (Taiwan Beer Basketball Team) partly because I believe they are a socially responsible organization.**	4.72 (5.48)	1.53 (1.57)	.88* (.91*)	19.21 (24.88)

*Note*. *p < .05; α: Cronbach’s alpha coefficient; AVE: average variance extracted; *M*: mean; SD: standard deviation; *λ*: standardized factor loading; t: t value; --: reference parameter. *χ*^2^/*df* = 1224.52 (1179.91)/539 (539) = 2.27 (2.18); RMSEA = 0.087 (0.079); NFI = 0.95 (0.97); NNFI = 0.97 (0.98); CFI = 0.97 (0.98); GFI = 0.72 (0.75); SRMR = 0.052 (0.052). Numbers presented outside (within) the parentheses refer to the results from Fubon Gladiators Baseball Team (Taiwan Beer Basketball Team). *N*_*1*_: sample size for Fubon Gladiators Baseball Team; *N*_*2*_: sample size for Taiwan Beer Basketball Team.

**Table 3:** Shared variances and AVEs for all constructs.

	PRCA	PRAE	HV	C	SV	E	NSE	SF	TI	POCA	POAE
**PRCA**	0.74 (0.82)	(0.88)	(0.24)	(0.33)	(0.33)	(0.28)	(0.16)	(0.29)	(0.50)	(0.44)	(0.51)
**PRAE**	0.82	0.79 (0.86)	(0.18)	(0.29)	(0.29)	(0.33)	(0.16)	(0.29)	(0.53)	(0.56)	(0.65)
**HV**	0.24	0.16	0.83 (0.79)	(0.75)	(0.53)	(0.27)	(0.32)	(0.46)	(0.15)	(0.17)	(0.21)
**C**	0.36	0.25	0.72	0.85 (0.82)	(0.82)	(0.30)	(0.24)	(0.42)	(0.30)	(0.22)	(0.23)
**SV**	0.34	0.26	0.57	0.88	0.90 (0.88)	(0.21)	(0.13)	(0.26)	(0.29)	(0.12)	(0.14)
**E**	0.11	0.10	0.16	0.07	0.07	0.57 (0.53)	(0.68)	(0.88)	(0.28)	(0.30)	(0.36)
**NSE**	0.02	0.02	0.16	0.05	0.04	0.65	0.65 (0.74)	(0.98)	(0.13)	(0.25)	(0.28)
**SF**	0.16	0.16	0.34	0.24	0.27	0.84	0.96	0.46 (0.51)	(0.27)	(0.33)	(0.39)
**TI**	0.40	0.34	0.34	0.54	0.50	0.06	0.15	0.21	0.91 (0.91)	(0.44)	(0.47)
**POCA**	0.38	0.28	0.24	0.33	0.32	0.21	0.34	0.24	0.39	0.80 (0.87)	(0.88)
**POAE**	0.46	0.50	0.27	0.34	0.37	0.16	0.27	0.28	0.42	0.72	0.84 (0.88)

Note. Numbers listed along the diagonal denote the AVE values. Numbers listed in the lower and upper triangles refer to the shared variances between constructs in Study 1 and Study 2, respectively. The numbers in parentheses are obtained from Study 2. PRCA: Pre-score of Cognitive Awareness; PRAE: Pre-score of Affective Evaluation; HV: Hedonic Value; C: Centrality; SV: Symbolic Value; E: Excitement; NSE: New Sport Event; SF: Sport Facility; TI: Team Identification; POCA: Post-score of Cognitive Awareness; POAE: Post-score of Affective Evaluation.

### Data analysis

To account for the participants’ initial perceptions of the research target, the pretest scores of CSR brand attitude were employed as a covariate. One-way analysis of covariance (ANCOVA) was used to test hypothesis H1; the dependent variable was the CSR brand attitude score, whereas independent variable was the method of message persuasion (central-route video, central-route picture, peripheral-route video, peripheral-route image, or control). Two-way ANCOVA was employed to test hypotheses H2–H4; the dependent variable was CSR brand attitude score, whereas the independent variables were involvement, sports team identification, sports fan curiosity, and method of message persuasion (central-route video, central-route text, peripheral-route video, peripheral-route image, or control). Involvement, sports team identification, and sports fan curiosity were subjected to nonhierarchical k-means cluster analysis for allocation of participants into high- and low-score groups to facilitate subsequent statistical analyses.

## Results

Using the ELM as a basis, this study explored the effects of persuasion strategy on consumers’ attitude toward sports organizations that fulfill CSR and their perceptions of the image of these organizations. The moderating effects of involvement, sports team identification, and sports fan curiosity were also examined.

### Study I: Fubon Guardians baseball team

The results of one-way ANCOVA were statistically significant (Table 4). For CSR perception, the central-route video group (M_Central Video_=5.17, SD_Central Video_=0.15) had significantly higher scores than the control group (M_Control Group_=4.55, SD_Control Group_=0.16, *p*=0.007), and the central-route text group (M_Central Text_=5.10, SD_Central Text_=0.15) had significantly higher scores than the control group (M_Control Group_=4.55, SD_Control Group_=0.16, *p*=0.01). This result partially supported H1, suggesting that message persuasion through the central-route video and central-route text on social media can effectively improve perception of CSR.

**Table 4:** ANCOVA summary based on different persuasion (Study 1).

Source	Type III SS	*df*	*MS*	*F*	*p*	*η*^2^
**Corrected model**	151.05	5	30.21	32.81**	<.001	0.47
**Intercept**	102.63	1	102.63	111.46**	<.001	0.37
**Attitude Toward CSR-Prescore**	139.03	1	139.03	151.00**	<.001	0.45
**Persuasion**	9.30	4	2.32	2.52*	0.04	0.05
**Error**	169.42	184	0.92			
**Total**	4939.95	190				
**Corrected total**	320.47	189				

*Note*. **p < .01;

*p<.05. Dependent variable: Attitude Toward CSR -Postscore. R^2^=0.47, Adj-R^2^=0.45.

In the two-way ANCOVA of different persuasion methods and involvement in baseball, the overall model was significant, and the interactive effect of persuasion method and involvement in baseball was significant at the level of .10 (Table 5). Under the condition of low involvement, significant differences in attitude toward CSR was found among different persuasions (F=3.13, p=0.02). Central route text (M_Central Text_=4.42, SD_Central Text_=0.26) is significantly greater than Control group (M_Control Group_=3.33, SD_Control Group_=0.30, *p*=0.008). Peripheral route video (M_Peripheral Video_=4.19, SD_Peripheral Video_=0.19) is significantly greater than Control group (M_Control Group_=3.33, SD_Control Group_=0.30, *p*=0.02). Central route video (M_Central Video_=4.58, SD_Central Video_=0.26) is significantly greater than Control group (M_Control Group_=3.33, SD_Control Group_=0.30, *p*=0.03). Central route video (M_Central Video_=4.58, SD_Central Video_=0.26) is significantly greater than Peripheral-route image group (M_Peripheral Image_=3.85, SD_Peripheral Image_=0.25, *p*=0.049). Furthermore, under the condition of high involvement, no significant difference in attitude toward CSR was found among different persuasions (F=1.36, p=0.25). This finding partially supported H2, indicating that persuasion through central and peripheral routes was more effective than the control group in terms of attitude toward a CSR brand among spectators with low involvement.

**Table 5:** ANCOVA for persuasion-involvement interaction (Study 1).

Source	Type III SS	*df*	*MS*	*F*	*p*	*η*^2^
**Corrected model**	174.96	10	17.49	21.52**	<.001	0.54
**Intercept**	115.75	1	115.75	142.39**	<.001	0.44
**Attitude Toward CSR-Prescore**	73.09	1	73.09	89.92**	<.001	0.33
**Persuasion**	11.76	4	2.94	3.61**	.007	0.07
**Involvement**	17.34	1	17.34	21.33**	<.001	0.10
**Persuasion x Involvement**	6.93	4	1.73	2.13*	.07	0.05
**Error**	145.51	179	0.81			
**Total**	4939.95	190				
**Corrected total**	320.47	189				

*Note*. **p < .01;

* *p* < .10. Dependent variable: Attitude Toward CSR -Postscore. R^2^=0.54, Adj-R^2^=0.52.

In two-way ANCOVA of persuasion method and sports team identification, the overall model was significant, and the interactive effect of persuasion method and sports team identification was nonsignificant (F=1.44, p=0.22; Table 6). H3 was thus not supported, implying that sports team identification did not moderate the relationship between message persuasion method and CSR perception.

**Table 6:** ANCOVA for persuasion-identification interaction (Study 1).

Source	Type III SS	*df*	*MS*	*F*	*p*	*η*^2^
**Corrected model**	177.81	10	17.78	22.31**	<.001	0.55
**Intercept**	121.13	1	121.13	151.98**	<.001	0.45
**Attitude Toward CSR-Prescore**	52.86	1	52.86	66.32**	<.001	0.27
**Persuasion**	7.35	4	1.83	2.30*	.06	0.04
**Identification**	22.31	1	22.31	27.99**	<.001	0.13
**Persuasion x Identification**	4.61	4	1.15	1.44	0.22	0.03
**Error**	142.66	179	0.79			
**Total**	4939.95	190				
**Corrected total**	320.47	189				

*Note*. **p < .01. Dependent variable: Attitude Toward CSR -Postscore. R^2^=0.55, Adj-R^2^=0.53.

In the two-way ANCOVA of persuasion method and sports fan curiosity, the overall model was significant, and the interactive effect of persuasion method and sports fan curiosity was significant (Table 7). Under the condition of low curiosity, significant differences in attitude toward CSR was found among different persuasions (F=5.06, p<0.001). Central route text (M_Central Text_=4.80, SD_Central Text_=0.21) is significantly greater than Control group (M_Control Group_=4.03, SD_Control Group_=0.20, *p*=0.012) and Peripheral-route image group (M_Peripheral Image_=4.10, SD_Peripheral Image_=0.18, *p*=0.016). Central route video (M_Central Video_=5.05, SD_Central Video_=0.16) is significantly greater than Peripheral route image group (M_Peripheral Image_=4.10, SD_Peripheral Image_=0.18, *p*<0.001), Peripheral route video (M_Peripheral Video_=4.28, SD_Peripheral Video_=0.17, p=0.002), and Control group (M_Control Group_=4.03, SD_Control Group_=0.16, *p*<0.001). Under the condition of high curiosity, no significant difference in attitude toward CSR was found among different persuasions (F=1.86, p=0.12). This finding supported H3, indicating that sports fan curiosity moderated the relationship between message persuasion method and CSR perception.

**Table 7:** ANCOVA for persuasion-curiosity interaction (Study 1).

Source	Type III SS	*df*	*MS*	*F*	*p*	*η*^2^
**Corrected model**	176.21	10	17.62	21.86**	<.001	0.54
**Intercept**	111.42	1	111.42	138.25**	<.001	0.44
**Attitude Toward CSR-Prescore**	107.77	1	107.77	133.73**	<.001	0.33
**Persuasion**	7.92	4	1.98	2.45**	.047	0.07
**Curiosity**	9.42	1	9.42	11.69**	.001	0.10
**Persuasion x Curiosity**	15.09	4	3.77	4.68**	.001	0.05
**Error**	144.26	179	0.80			
**Total**	4939.95	190				
**Corrected total**	320.479	189				

*Note*. **p < .01. Dependent variable: Attitude Toward CSR -Postscore. R^2^=0.55, Adj-R^2^=0.52.

### Study II: Taiwan Beer Basketball Team

The results of one-way ANCOVA were statistically significant (Table 8). The central-route video (M_Central Video_=5.63, SD_Central Video_=0.13, *p*<0.001), central-route text (M_Central Text_=5.95, SD_Central Text_=0.13, *p*<0.001), peripheral-route video (M_Peripheral Video_=5.90, SD_Peripheral Video_=0.13, *p*<0.001), and peripheral-route picture (M_Peripheral Image_=5.64, SD_Peripheral Image_=0.13, *p*<0.001) groups had significantly higher CSR perception scores than the control group (M_Control Group_=5.10, SD_Control Group_=0.13). This result supported H1, suggesting that message persuasion through both the central and peripheral routes via social media can effectively improve CSR perception.

**Table 8:** ANCOVA summary based on different persuasion (Study 2).

Source	Type III SS	*df*	*MS*	*F*	*p*	*η*^2^
**Corrected model**	235.16	5	47.03	65.22*	<.001	0.62
**Intercept**	85.50	1	85.50	118.57*	<.001	0.37
**Attitude Toward CSR-Prescore**	194.56	1	194.56	269.82*	<.001	0.58
**Persuasion**	17.79	4	4.44	6.16*	<.001	0.11
**Error**	139.89	194	0.72			
**Total**	6759.55	200				
**Corrected total**	375.05	199				

*Note*. *p < .05. Dependent variable: Attitude Toward CSR -Postscore. R^2^=0.62, Adj-R^2^=0.61.

In the two-way ANCOVA of persuasion method and involvement in basketball, the overall model was significant, and the interactive effect of persuasion method and involvement in basketball was significant (Table 9). Under the condition of low involvement, significant differences in attitude toward CSR was found among different persuasions (F=5.82, p=0.001). Central route text (M_Central Text_=5.50, SD_Central Text_=0.25, p<0.001), Central route video (M_Central Video_=5.11, SD_Central Video_=0.32, p=0.005), Peripheral route image group (M_Peripheral Image_=4.93, SD_Peripheral Image_=0.25, *p*=0.006), and Peripheral route video (M_Peripheral Video_=5.74, SD_Peripheral Video_=0.35, p<0.001) is significantly greater than Control group. However, under high involvement conditions, message persuasion method did not have a significant effect (F=1.84, p=0.12). This finding support H2, indicating that persuasion through central and peripheral routes was more effective than the control treatment in terms of attitude toward a CSR brand among spectators with low involvement.

**Table 9:** ANCOVA for persuasion-involvement interaction (Study 2).

Source	Type III SS	*df*	*MS*	*F*	*p*	*η*^2^
**Corrected model**	243.65	10	24.36	35.04**	<.001	0.65
**Intercept**	79.37	1	79.37	114.16**	<.001	0.37
**Attitude Toward CSR-Prescore**	136.90	1	136.90	196.91**	<.001	0.51
**Persuasion**	24.59	4	6.14	8.84**	<.001	0.15
**Involvement**	0.003	1	0.003	0.004	0.95	<.001
**Persuasion x Involvement**	8.47	4	2.11	3.04*	0.01	0.06
**Error**	131.39	189	0.69			
**Total**	6759.55	200				
**Corrected total**	375.05	199				

*Note*. **p < .01;

* *p* < .05. Dependent variable: Attitude Toward CSR -Postscore. R^2^=0.65, Adj-R^2^=0.63.

In the two-way ANCOVA of persuasion method and sports team identification, the overall model was significant, as was the interactive effect of persuasion method and sports team identification (F=5.28, p<0.001; Table 10). Under the condition of low team identification, significant differences in attitude toward CSR was found among different persuasions (F=5.39, p=0.01). Central route text (M_Central Text_=5.11, SD_Central Text_=0.32, p<0.001), Central route video (M_Central Video_=4.33, SD_Central Video_=0.33, p=0.045), Peripheral route image (M_Peripheral Image_=4.51, SD_Peripheral Image_=0.30, p=0.012), and Peripheral route video (M_Peripheral Video_=5.77, SD_Peripheral Video_=0.64, p=0.002) are significantly greater than Control group (M_Control Group_=3.40, SD_Control Group_=0.29). However, under strong sports team identification conditions, message persuasion method did not have a significant effect (F=1.33, p=0.26). This finding supported H3, indicating that persuasion through the central and peripheral routes was more effective than the control treatment in terms of attitude toward CSR brands for spectators with low team identification.

**Table 10:** ANCOVA for persuasion-identification interaction (Study 2).

Source	Type III SS	*df*	*MS*	*F*	*p*	*η*^2^
**Corrected model**	255.01	10	25.50	40.15**	<.001	0.68
**Intercept**	91.36	1	91.36	143.86**	<.001	0.43
**Attitude Toward CSR-Prescore**	91.40	1	91.40	143.91**	<.001	0.43
**Persuasion**	25.96	4	6.49	10.22**	<.001	0.17
**Identification**	2.33	1	2.33	3.67*	0.057	0.01
**Persuasion x Identification**	13.43	4	3.35	5.28**	<.001	0.10
**Error**	120.03	189	0.63			
**Total**	6759.55	200				
**Corrected total**	375.05	199				

*Note*. **p < .01;

* *p* < .10. Dependent variable: Attitude Toward CSR -Postscore. R^2^=0.68, Adj-R^2^=0.66.

In the two-way ANCOVA of persuasion method and sports fan curiosity, the overall model was significant; however, the interactive effect of persuasion method and sports fan curiosity was nonsignificant (F=0.36, p=0.83, Table 11). H4 was thus rejected, indicating that sports fan curiosity did not moderate the relationship between message persuasion method and CSR perception.

**Table 11:** ANCOVA for persuasion-curiosity interaction (Study 2).

Source	Type III SS	*df*	*MS*	*F*	*p*	*η*^2^
**Corrected model**	245.72	10	24.57	35.91**	<.001	0.65
**Intercept**	94.33	1	94.33	137.86**	<.001	0.42
**Attitude Toward CSR-Prescore**	135.39	1	135.39	197.86**	<.001	0.51
**Persuasion**	17.43	4	4.35	6.36**	<.001	0.11
**Curiosity**	9.84	1	9.84	14.39**	<.001	0.07
**Persuasion x Curiosity**	1.00	4	0.25	0.36	0.83	0.01
**Error**	129.32	189	0.68			
**Total**	6759.55	200				
**Corrected total**	375.05	199				

*Note*. **p < .01. Dependent variable: Attitude Toward CSR -Postscore. R^2^=0.65, Adj-R^2^=0.63.

## Discussion

### General discussion

Study I showed that communication through central route increased consumer CSR perception scores. Persuasion through central route involves a considerable amount of attitude information. When individuals process information through the central route, they are likely to diligently consider and evaluate the content of the information; therefore, attitude formation or attitude change induced through the central route is relatively more enduring [48]. Study II showed that communication through both the central and peripheral routes increased consumer CSR perception scores. Compared with the central route, the peripheral route is characterized by low to nonexistent relevance between the message and message recipient; individuals process the message by relying on other heuristic or simple cues rather than the main argument of the message. Attitude formation or attitude change induced through the peripheral route is relatively temporary and unenduring [20]. This study demonstrated that in a professional sports setting, communication on social media was effective through both the central and peripheral routes.

Studies I and II revealed that involvement moderated the relationship between message persuasion method and CSR perception. Under low involvement conditions, both the central and peripheral routes improved consumer CSR perception scores. However, under high involvement conditions, the various persuasion methods were found to have nonsignificantly different effects. Although studies indicated that when consumers with higher involvement are judging the CSR image of a company, they take more information into consideration, signifying that they elaborate more and spend more time processing CSR information [11, 54]. Nevertheless, highly involved consumers believe that sporting activity has high personal relevance and are likely already aware of the CSR-related activities conducted by the professional sports teams considered in this study. Hence, under high involvement conditions, persuasion method did not significantly influence CSR perception scores. Conversely, under low involvement conditions, consumers do not believe that sporting activity has high personal relevance and therefore are likely not aware of the CSR activities conducted by a sports team. In this situation, a strategy of communication through both the central and peripheral routes is thus likely to improve consumer CSR perception scores. This result echoes the findings of related empirical studies that some consumers identify with a firm’s CSR practice and in turn purchase from the firm, whereas other consumers are indifferent to the firm’s practice; consumer response is thus dependent on involvement. Under the impact of COVID-19, the quarantine policies of sports franchises may influence sporting event attendance. Therefore, sports teams can communicate with current and prospective spectators through social media using central- and peripheral-route persuasion to attract individuals with no involvement or low involvement in sports.

The moderating effect of sport team identification the relationship between method of persuasion and CSR perception was nonsignificant in Study I and significant in Study II. Under high sport team identification conditions, persuasion method exerted a nonsignificant effect on CSR perception. This finding may have been because the strength of sports team identification affects the perceptions, attitude, and consumption behavior of sports consumers [61], and when message recipients are more certain of their attitude toward a target message, information processing is likely to be biased [62]. Additionally, individuals with higher sports team identification tend to use media more frequently to follow their sports team [24], are more inclined to purchase team-related merchandise, and spread positive word of mouth for the sports teams they support [63, 64]. By contrast, under weak sports team identification, both the central and peripheral route persuasion strategies are effective. A possible explanation for this result is as follows. Weak identification with a sports team implies that consumers have relatively weak psychological connection to the sports team; therefore, they are unlikely to attempt to understand the team’s CSR activities and in turn are unaware of the team’s CSR efforts. When an opportunity presents itself to introduce information on a sports team’s CSR activities, strategies under the central and peripheral routes can provide consumers with information regarding the team’s CSR activities, improving consumers’ CSR attitude.

The moderating effect of sports fan curiosity on the relationship between method of persuasion and CSR perception was significant in Study I and nonsignificant in Study II. Under high sports fan curiosity conditions, persuasion method exerted a nonsignificant effect on CSR perception. Thus, curious sports fans exhibit a more positive attitude toward their favorite sports team’s CSR, which is why their attitude toward CSR did not differ significantly depending on the persuasion strategy. Litman and Speilberer defined individual curiosity as reaction and desire that induce exploratory behavior [65]. Curious consumers should have more pronounced behavior and greater motivation to explore the CSR activities of their favorite sports teams. Hence, these consumers generally exhibit more positive attitude toward a team’s CSR, which is reflected by the nonsignificant effects of the various persuasion strategies. Conversely, under low sports fan curiosity conditions, the central-route text group had significantly higher scores than the control and peripheral-route picture groups for CSR perception. This result was possibly because relatively low curiosity signifies a lower likelihood to attempt to understand a sports team’s CSR activities, thereby leading to ignorance of the team’s CSR efforts. Communication of textual information through the central route gives consumers information regarding the sports team’s CSR activities. Consumers in this experimental group had significantly higher CSR attitude scores than the control group, who did not receive information on the sports team’s CSR activities, and the peripheral-route picture group.

This study has implications for both research and practice. Regarding research implications, this study contributes to the literature on CSR in sport by using the ELM as a theoretical framework to explore the effects of various persuasion strategies on the attitude toward the CSR image of professional sports teams of consumers who use social media. In addition, this study determined the moderating effects of involvement, sports team identification, and sports fan curiosity, providing further understanding of communication strategies in sport. Regarding implications for practice, professional sports teams aiming to improve consumer attitude toward their CSR image could adopt a central or peripheral route strategy to communicate the team’s CSR-related information to consumers with low involvement, weak sports team identification, and low sports fan curiosity.

### Limitations and directions for future study

This study has several limitations. First, random sampling is difficult to conduct in practice, which was why this study did not collect a sample through random sampling. Future studies could attempt to overcome the difficulties of random sampling. Second, this study conducted two experiments, one on a baseball team (Study I) and the other on a basketball team (Study II), to ensure the generalizability of the results. However, this study was still conducted in only a single region. Scholars are recommended to conduct empirical research in different countries or settings to ensure generalizability. Finally, the moderating effect of sports fan curiosity on the relationship between persuasion method and CSR perception was significant in Study I but nonsignificant in Study II, showing that this moderating effect should be verified. The moderating effect and role of sports team identification also require verification in the future.

## Conclusion

The fulfillment of CSR is none other than firms fulfilling their civic role and duty to society. CSR reflects their hope to improve both their corporate image and business performance through social responsibility activities. The key to achieving this goal is CSR communication. Although studies have explored the effect of communicating CSR information to stakeholders, there remains a dearth of academic research on this issue in the sport management setting. Social media has become one of the main channels through which information is communicated between people or between organizations and individuals. This study determined the moderating effects of involvement, sports team identification, and sports fan curiosity, contributing to the literature on communication strategies in sport. In addition, professional sports teams aiming to improve consumer attitude toward their CSR image could adopt a central route or peripheral route strategy to communicate the team’s CSR-related information to consumers with low involvement, weak sports team identification, and low sports fan curiosity.

## Supporting information

S1 Dataset (SAV)

S2 Dataset (SAV)

S1 File (XLSX)
